# Successful Expansion but Not Complete Restriction of Tropism of Adeno-Associated Virus by *In Vivo* Biopanning of Random Virus Display Peptide Libraries

**DOI:** 10.1371/journal.pone.0005122

**Published:** 2009-04-09

**Authors:** Stefan Michelfelder, Johannes Kohlschütter, Alexandra Skorupa, Sabrina Pfennings, Oliver Müller, Jürgen A. Kleinschmidt, Martin Trepel

**Affiliations:** 1 Department of Hematology and Oncology, University of Freiburg Medical Center, Freiburg, Germany; 2 Institute for Molecular Medicine and Cell Research, Freiburg, Germany; 3 University Medical Center Hamburg-Eppendorf, Department of Oncology and Hematology, Hamburg, Germany; 4 University of Heidelberg, Internal Medicine III, Heidelberg, Germany; 5 Deutsches Krebsforschungszentrum, Heidelberg, Germany; Instituto Butantan, Brazil

## Abstract

Targeting viral vectors to certain tissues *in vivo* has been a major challenge in gene therapy. Cell type-directed vector capsids can be selected from random peptide libraries displayed on viral capsids *in vitro* but so far this system could not easily be translated to *in vivo* applications. Using a novel, PCR-based amplification protocol for peptide libraries displayed on adeno-associated virus (AAV), we selected vectors for optimized transduction of primary tumor cells *in vitro*. However, these vectors were not suitable for transduction of the same target cells under *in vivo* conditions. We therefore performed selections of AAV peptide libraries *in vivo* in living animals after intravenous administration using tumor and lung tissue as prototype targets. Analysis of peptide sequences of AAV clones after several rounds of selection yielded distinct sequence motifs for both tissues. The selected clones indeed conferred gene expression in the target tissue while gene expression was undetectable in animals injected with control vectors. However, all of the vectors selected for tumor transduction also transduced heart tissue and the vectors selected for lung transduction also transduced a number of other tissues, particularly and invariably the heart. This suggests that modification of the heparin binding motif by target-binding peptide insertion is necessary but not sufficient to achieve tissue-specific transgene expression. While the approach presented here does not yield vectors whose expression is confined to one target tissue, it is a useful tool for *in vivo* tissue transduction when expression in tissues other than the primary target is uncritical.

## Introduction

Efficient and specific delivery of therapeutic genes to the tissue of interest is a paramount and so far unsolved issue in gene therapy. Among the available viral vectors for gene delivery, adeno-associated virus (AAV) has gained particular attention. The low frequency of random integration into the genome [1] and the moderate immune response make AAV an attractive basis for gene therapy vector design [2], [3]. No substantial safety issues have been encountered in a number of clinical trials involving AAV vectors [1]. Like in almost all other gene therapy vectors, the tropism of AAV-2 derived vectors limits its use for the gene transduction of certain tissues especially when vectors are delivered systemically. This may partly be circumvented by using AAV serotypes with an *in vivo* gene transduction pattern most closely fitting the needs of the application [4]. Also, the tropism of AAV capsids may be changed by combining parts of the natural serotype diversity (reviewed in [5]). Alternatively or in addition, peptides mediating binding to the cell type of interest can be identified by random phage display library screening and subsequently be introduced into an AAV capsid region critical for receptor binding [6], [7], [8], [9], [10], [11], [12]. Such peptide insertions into or other mutational manipulations of the heparin binding domain adjacent to VP capsid protein position R588 can abrogate the natural tropism of AAV-2 capsids to heparan sulfate proteoglycane (HSPG)-expressing cells and result in de-targeting from the liver *in vivo* [13], [14], [15]. The identification of numerous tissue-directed peptide ligands during the last decade [16], [17], [18], [19], [20], [21], [22], [23], [24], [25], [26], [27], [28], [29], [30], [31], [32] would seem to supply almost unlimited potential for the introduction of ligands into AAV capsids to establish targeted gene delivery *in vivo*. This approach has indeed been reported to be successful using certain peptides [10], [11], [12]. Yet, our own experience has been, that for many peptides cell tropism changes or gets lost after inserting them into the AAV capsid (author's unpublished observation). This may be due to a number of reasons. First and foremost, the peptide's conformation may change unpredictably when incorporated into the structural AAV capsid context, leading to a reduced receptor-ligand affinity and specificity. Further, peptides isolated by phage display screenings are commonly selected based on receptor binding but not on subsequent internalization, nuclear transfer, and transgene expression. To overcome these obstacles, a screening system based on random peptide libraries displayed directly on AAV capsids has recently been developed were the AAV library particles are amplified based on binding, uptake and viral gene expression in the target cell via adenoviral helper co-infection [14], [33]. Capsid mutants efficiently transducing various different cell types have been isolated from such libraries by biopanning on the cells of interest [14], [33], [34], [35], [36], [37]. Even though numerous AAV capsid variants have been isolated by AAV library screenings, comparative gene expression analyses of such modified AAV derived vectors have not been performed *in vivo* and, despite the obvious importance of the question, it remains open for most of these vectors whether or not a retargeting after systemic administration *in vivo* occurs.

Vector targeting *in vivo* faces several hurdles which are not present *in vitro* and the mechanisms that determine a vectors tropism and its gene transduction properties *in vivo* are as yet poorly understood. While *in vivo* biodistribution of a vector is to a considerable part defined by clearance, its gene transduction properties are rather dependent on receptor binding, cellular uptake, nuclear transfer, and transgene expression. Thus, major hurdles for receptor targeted gene transfer *in vivo* are to improve specific ligand-receptor interactions under circulation conditions as well as to overcome host-anti-vector immune reactions, rapid vector clearance from the circulation by the reticuloendothelial system, and endothelial cell layers as well as the extracellular matrix acting as physical barriers [38]. Taking these considerations into account, *in vivo* biopanning of random AAV peptide libraries seems to be more appropriate to select for tissue directed gene vectors than mere tissue culture-based approaches. Among the limitations faced by *in vivo* AAV display library selection is the difficulty to rescue and amplify tissue-targeted library viruses for multiple selection rounds as the amplification systems used *in vitro* are based on adenoviral superinfection and can therefore not easily be applied in living animals.

In this study, we set out to isolate tissue-directed AAV capsids using murine breast cancer and lung tissue as prototype targets. We established a novel adenovirus-free PCR based screening approach that amplifies tissue-targeted library viruses and therefore allows for multiple AAV library screening rounds after systemic application *in vivo*. We further analyzed gene transduction properties of the isolated capsid variants. While the selected vectors indeed transduced their target tissue orders of magnitude better than unselected vectors, we almost invariably observed unintended transduction of heart tissue. These results show that modification of the HSPG-binding capsid domain of AAV vectors by targeting peptide insertion is necessary but not sufficient to achieve completely tissue-specific transgene expression while this technical approach may be appropriate when expansion rather than restriction of AAV tropism to the tissue of interest is needed. Our findings therefore broaden the functional understanding of AAV-2 vectors, particularly when selected from random AAV display peptide libraries.

## Materials and Methods

### Cells and cell culture

293T cells (kindly provided by David Baltimore, California Institute of Technology, Pasadena, CA), were maintained in Dulbecco's Modified Eagle's medium (DMEM; Invitrogen, Carlsbad, CA) containing 1% penicillin/streptomycin solution (Invitrogen) and 10% fetal calf serum (FCS; Biochrom, Berlin, Germany). Primary murine breast cancer cells were obtained from tumors growing in female transgenic FVB mice expressing the polyoma middle T antigen under the control of the mouse mammary tumor virus promoter [39], [40] as previously described [41]. Briefly, tumors were cut into small pieces and digested for 1 h at 37°C in collagenase 2 solution (Biochrom), dissolved in PBS, 10% 2 mM MgCl_2_/CaCl_2_ and 10% BSA. The cell suspension was passed through 100 µm and 40 µm cell strainers, washed twice with PBS, and cultured in Iscove's Modified Dulbecco's Medium (IMDM; Invitrogen) containing 10% fetal bovine serum, 10% horse serum, 1% penicillin/streptomycin, and 1.25 µg/ml amphotericin B (Invitrogen). All cells were cultured in a humidified atmosphere at 37°C and 5% CO_2_. Immunodetection using a pan-cytokeratin antibody (Sigma) revealed more than 95% of cytokeratin-positive tumor cells after 24 hours in culture.

### Animals and tumor staging

All procedures involving animals were performed according to the Guide for the Care and Use of Laboratory Animals published by the US National Institutes of Health (NIH Publication No. 85-23, revised 1996) and the German Animal Protection Code. We used a transgenic breast cancer mouse model induced by the polyoma middle T antigen (PymT) under control of the mouse mammary tumor virus promoter. The mouse strain FVB/N-TgN(MMTVPyVT)634-Mul (PymT) was purchased from Jackson Laboratory (Bar Harbor, ME). Genotyping was performed by polymerase chain reaction (PCR) as described by Jackson Laboratory (www.jax.org). Starting at the age of 30 days, transgenic female mice were palpated weekly for early detection of mammary tumors. The animals were anesthetized by intraperitoneal injection of 100 mg/kg body weight 10% ketamine hydrochloride (115.34 mg/ml; Essex, Munich, Germany) and 5 mg/kg body weight 2% xylazine hydrochloride (23.32 mg/ml; Bayer, Leverkusen, Germany).

### AAV peptide library biopanning in vitro and in vivo

**Figure 1:**
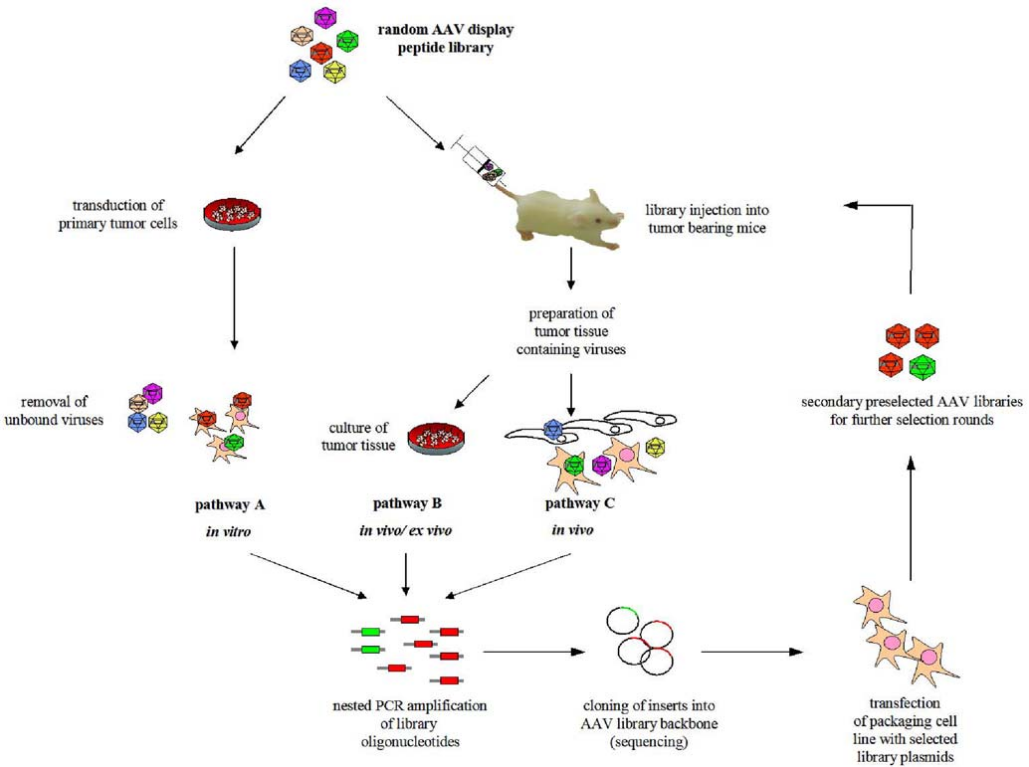
Pathways used for selection of targeted viral capsids by screening random AAV display peptide libraries. For all selection pathways, genomic DNA containing *cap* gene fragments from internalized library viruses was extracted from the target cells or tissue. Library inserts were amplified by nested PCR and cloned back into the AAV library backbone plasmid pMT-202-6. The resulting pre-selected plasmid library was used to produce a secondary AAV library by transfection into 293T cells and subsequent superinfection with Ad5. Pre-selected AAV libraries were re-subjected to selection on the target cells *in vitro* or the target tissue *in vivo*. Preceding the amplification step, the library selection was done according to one of the following three pathways: Pathway A, *in vitro* selection: A random AAV display peptide library was incubated on primary breast cancer dissociation cultures derived from female tumor-bearing PymT mice. Non-internalized AAV library particles were removed by extensive washing followed by trypsin digestion prior to DNA extraction and AAV insert amplification. Pathway B, *in vivo*/*ex vivo* selection: A random AAV display peptide library was injected intravenously into female tumor-bearing PymT mice. After 24 hours, primary tumor cells of the injected mouse were prepared as in pathway A and grown *ex vivo* for 96 hours prior to DNA extraction and AAV insert amplification. Pathway C, *in vivo* selection: A random AAV display peptide library was injected as in pathway B in tumor-bearing mice (for selection of tumor-homing AAV) or wild-type mice (for selection of lung homing AAV), respectively. After 48 hours, the target tissue (tumor or lung, respectively) was removed and lysed, and DNA was extracted for AAV insert amplification.

A random X_7_ AAV display peptide library (random insert introduced at position R588 VP1 capsid protein numbering) with a diversity of 2×10^8^ random clones (determined at the cloned plasmid level) was produced using a three-step protocol as described previously [14], [35]. For *in vitro* biopanning (Figure 1, pathway A), 2×10^6^ primary PymT breast cancer cells were incubated with the AAV library at a multiplicity of infection (MOI) of 1.000 vector genomes (vg)/cell in selection round 1, 500 vg/cell in round 2, and 100 vg/cell in round 3. After 96 hours, unbound AAV library particles were removed by 3 washing steps in PBS. Surface-bound library viruses were detached by trypsin digestion for 20 minutes and subsequent washing. Previous work had shown that this additional trypsin digest is essential to enrich internalizing clones for improved transduction of the target cells (M.T., unpublished observation). Whole cellular DNA was extracted using the QIAamp Tissue Kit (Qiagen, Hilden, Germany). The random oligonucleotides contained in AAV library particles internalized into tumor cells were amplified by PCR using the primers 5′-GGTTCTCATCTTTGGGAAGCAAG-3′ and 5′-TGATGAGAATCTGTGGAGGAG-3′. For *in vivo*/*ex vivo* biopanning of AAV peptide libraries (Figure 1, pathway B), 1×10^10^ vg of an AAV library for selection round 1, or 2×10^8^ to 2×10^9^ vg per animal for round 2–4 were injected into the tail vein of female PymT transgenic mice bearing palpable breast tumors. After 24 hours, primary breast cancer cells were prepared as described above and grown *in vitro* for 96 hours. Oligonucleotide inserts of targeted AAV library particles were amplified by nested PCR using whole cellular DNA as template. Primers were 5′-ATGGCAAGCCACAAGGACGATG-3′ and 5′- CGTGGAGTACTGTGTGATGAAG-3′ for the first PCR and 5′-GGTTCTCATCTTTGGGAAGCAAG-3′ as well as 5′-TGATGAGAATCTGTGGAGGAG-3′ for the second PCR. Pure *in vivo* library biopanning (Figure 1, pathway C) was performed along the same lines, except that the circulation time was 48 hours and that DNA extraction from the tumor tissue was done without prior *ex vivo* growth of the cells. To select for lung homing AAV, libraries were injected into the tail vein of 6-week-old female PycB/FVB wild-type mice (n = 2 animals per selection round) as described for tumor selections (Figure 1, pathway C). DNA of whole lung tissue extracts from two animals was extracted, pooled and used as template to amplify the random oligonucleotide of lung-homing AAV. We varied the time of AAV blood circulation before lung harvest in 2 alternative selection approaches (5 minutes followed by a perfusion step, 48 hours in round 1, 48 hours or 6 days for round 2, and 6 days for round 3 to 4). For all selections, PCR products were analyzed by agarose gel electrophoresis to verify correct size, digested with *Bgl*I and cloned into the *Sfi*I-digested pMT-202-6 library backbone plasmid [14], [35]. Cloned AAV library plasmids were transformed into electrocompetent *E. coli* DH5-α (Invitrogen) using the Gene Pulser (Bio-Rad, Hercules, CA). Randomly assigned clones were sequenced using the reverse primer 5′-CAGATGGGCCCCTGAAGGTA-3′. For production of pre-selected AAV peptide libraries, 2×10^8^ 293T cells were transfected with the library plasmids at a ratio of 25 plasmids/cell using Qiagen's PolyFect reagent. pUC18 (Invitrogen) served as carrier DNA. Two hours after transfection, 293T cells were superinfected with wild-type adenovirus type 5 (Ad5, supplied by the Laboratoire de Thérapie Génique, Nantes, France) at an MOI of 5 infectious particles/cell for library particle amplification. After 48 h, or when cell lysis became apparent, cells were detached from the culture dish in PBS-MK (140 mM NaCl, 5.5 mM KCl, 8 mM Na_2_HPO_4_, 1.5 mM KH_2_PO_4_, 1 mM MgCl_2_) and pooled with supernatants. AAV library particles were harvested by cell lysis via three freeze-thaw cycles. Cellular DNA was removed by incubation with benzonase (Sigma) at 50 U/ml lysate at 37°C for 30 min, followed by Ad5 inactivation at 55°C for 30 min. Viral library preparations were purified using the iodixanol gradient centrifugation method as previously described [42], [43]. The 40% iodixanol fraction containing the purified AAV viruses was stored at −80°C until further use.

### Production of capsid modified recombinant AAV vectors

Recombinant AAV (rAAV) vectors displaying selected peptide sequences were generated by cloning the oligonucleotide inserts into the pXX2-187 plasmid (a derivative of the library backbone plasmid pMT-187-0-3 without ITRs) [34], [35]. 293T packaging cells were transfected with the modified pXX2-187 (or pXX2 for wild-type AAV-2 capsid controls), pXX6 [44], and a plasmid carrying a reporter gene or a toxic transgene of interest using PolyFect (Qiagen). Packaged reporter genes included the luciferase (luc) gene in the plasmid pUF2-CMV-luc [34]. The HSV thymidine kinase mutant SR39 [45] was used as a cytotoxic suicide gene. Cells were harvested 96 hours after transfection, and vectors were purified by iodixanol gradient centrifugation as described above.

### AAV titration and evaluation of vector homing and serum distribution

The AAV capsid titers were determined as described [46] by ELISA (Progen, Heidelberg, Germany). The genomic titers of recombinant AAV vectors and AAV libraries were determined by quantitative PCR using the Absolute SYBR Green fluorescein master mix (Abgene, Epsom, UK) and the MyiQ cycler (Bio-Rad) as previously described [47], [48]. Vectors were quantified using the forward primer 5′-GGCGGAGTTGTTACGACAT-3′ and the reverse primer 5′-GGGACTTTCCTACTTGGCA-3′ specific for the CMV promoter sequence. The genomic titer of AAV libraries was determined using the forward primer 5′-GCAGTATGGTGTATCTACCAA-3′ and the reverse primer 5′-GCCTGGAAGAACGCCTTGTGT-3′ specific for the AAV *cap* gene. Real-time PCR was done in 20 µl with 0.3 µM for each CMV primer, or 0.4 µM for each AAV primer, respectively, according to the manufacturer's protocol (Abgene). For CMV primers, annealing temperature was 64°C for 15 seconds. For AAV primers, annealing temperature was 61°C for 30 seconds. Fluorescence was measured at the end of each annealing phase. A standard curve for quantification was generated by serial dilutions of the respective vector plasmid DNA. Calculations were done using MyIQ analysis software (Bio-Rad). For quantification of vectors homing to lung tissue, 5×10^10^ capsid-modified rAAV-luciferase vectors were injected into the tail vein of female PycB/FVB wild-type mice (n = 3 per group). After 8 d, lung tissue was removed. Whole DNA was extracted using the DNeasy tissue kit (Qiagen) and quantified using a 2100Pro spectrophotometer (Amersham Pharmacia Biotech, Uppsala, Sweden). For real-time PCR, 500 ng of extracted genomic DNA were used as template to amplify vector specific DNA using CMV primers as described above. To determine the amount of circulating AAV library or wild-type viruses in the blood, 1×10^10^ vg were injected into the tail vein of PycB/FVB wild type mice. Blood was obtained at indicated time points and centrifuged for 2 minutes at 10,000 rpm. Cell-free serum was diluted 1∶100 in ddH_2_O and used as template for real-time PCR using AAV specific primer pairs as described above.

### Luciferase gene transduction

To analyze luciferase gene transduction *in vitro*, 2×10^4^ cells per well were seeded in 24-well plates or 5×10^3^ cells per well in 96-well plates and incubated with AAV-luciferase vectors at an MOI of 10,000 vg/cell for 72 h. For *in vivo* gene transfer, 5×10^10^ vg of rAAV-luciferase vectors were injected into the tail vein of anesthetized animals. After 8 or 28 days, respectively, the target tissue and representative control tissues were removed, snap frozen in liquid nitrogen, and stored at −80°C. Frozen tissue samples and cell lysates were homogenized in reporter lysis buffer (RLB, Promega, Madison, WI) and luciferase reporter gene activity was determined in a luminometer (Centro LB 960, Berthold Technologies, Bad Wildbad, Germany) using Promega's luciferase assay according to the manufacturer's instructions. If required, values were normalized to protein levels in each probe determined by Bradford assay (Bio-Rad).

### Suicide gene transfer and toxicity assay

Cells were seeded at 5×10^3^ per well in 96-well plates and transduced with rAAV-SR39 vectors at an MOI of 10,000 vg/cell. After two cycles of 10 µM ganciclovir (GCV) treatment (24 hours and 72 hours after transduction), the number of viable cells was assessed as described [49], [50]. Cells were incubated with medium containing 500 µg/ml MTT (Invitrogen) for 4 h. Subsequently, absorbance of formazan crystals dissolved in SDS/HCl was measured at 570 nm in a SpectraMAX microplate reader (Molecular Devices, Sunnyvale, CA).

### Statistics

Statistical analysis was performed using the GraphPad Prism program 3.0 (GraphPad Software, San Diego, CA). Parametric data were analyzed by one-way analysis of variance followed by a Bonferroni post test. Non-parametric data were analyzed by a Kruskal-Wallis test followed by a Dunn's post test. p values <0.05 were considered significant.

## Results

### PCR-based screening of a random AAV display peptide library on primary breast cancer cells yields enrichment of specific peptide motifs

To isolate AAV-2 capsids for targeted gene transfer in primary breast cancer cells of transgenic PymT mice, we prepared tumor cells and screened an X_7_ random AAV display peptide library *in vitro* along the lines of pathway A in Figure 1 by which internalized AAV library particles are amplified based on PCR amplification of their random oligonucleotide insert. The *cap* gene region containing the oligonucleotide insert of AAV recovered from breast cancer cells after each round of selection was amplified by nested PCR and correct size of the amplification product was verified by agarose gel electrophoresis (data not shown). The insert was cloned back into the library backbone plasmid pMT202-6 and the diversity of transformed library plasmids was at least 1×10^5^ clones for such secondary libraries in this and subsequent selections (data not shown). New pre-selected AAV particle libraries were obtained by transfection of 293T cells with the generated secondary plasmid library in limiting dilution technique (25 library plasmid molecules per producer cell) to minimize the production of chimeric AAV library particles or mismatch of packaged DNA and displayed peptide due to uptake of multiple library genomes in one producer cell. The titers obtained with this approach were sufficient for further selection rounds (data not shown). To increase the stringency of selection, MOIs of AAV libraries were decreased from 500 vg/cell to 100 vg/cell in rounds two and three, respectively. Sequence analysis showed enrichment of several clones after two rounds of selection compared to round 1, functionally validating our novel selection method. Peptide sequences found after round 1 were RGDLGLS, RGDMSRE, DGLGRLV, and DRSPLSL. After three rounds of selection, RGDLGLS and RGDMSRE were the dominant clones (Table 1). Both peptides share the sequence motif RGDXXXX.

**Table 1:** Peptides enriched after PCR-based *in vitro* selection (pathway A) of AAV peptide libraries on primary breast cancer cells

Peptide sequence a	Charge pattern b	Frequency in selection round c		
		round 1	round 2	round 3
RGDLGLS	+y-yyyx	-	3/10	6/9
RGDMSRE	+y-yx+-	-	1/10	3/9
DGLGRLV	-yyy+yy	-	3/10	-
DRSPLSL	-+xyyxy	1/6	2/10	-

a single letter code; shared amino acid patterns are highlighted in colored letters

b charge pattern of amino acid side chains: +, positively charged; − negatively charged; x, uncharged polar; y, nonpolar.

c observed frequency relative to overall number of sequenced clones

### Selected AAV capsids efficiently transduce primary murine breast cancer cells

**Figure 2:**
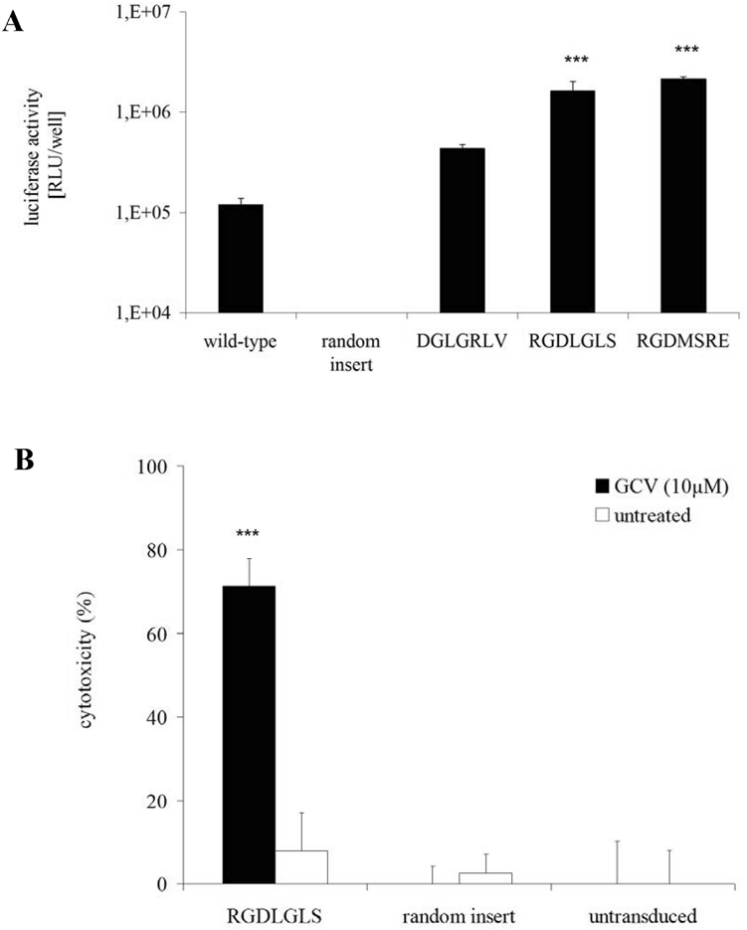
Vector capsids selected from random AAV display peptide libraries for targeted gene transfer in primary breast cancer cells. A: Primary PymT breast cancer cells were transduced by recombinant AAV-2 luciferase reporter gene vectors displaying the selected capsid peptide inserts RGDLGLS, RGDMSRE, or DGLGRLV, respectively. Capsids with no (wild-type) or random peptide insert (VRRPRFW) were used as controls. Transduction efficiency was determined after 72 hours by luciferase assay. Luciferase activities are shown in relative light units (RLU) per well. Data represent mean values ± standard deviation (SD) from one representative experiment (out of three) in triplicates (*** = p<0.001 compared to wild-type and random insert controls). B: Breast cancer cell-targeted therapeutic suicide gene transfer using selected capsid mutants. Primary PymT cells were transduced using rAAV-SR39 vectors displaying RGDLGLS or a randomly selected control peptide (VRRPRFW). Four days after initiation of ganciclovir (GCV) treatment, cytotoxic effects were evaluated by MTT assay. Values are shown in % cytotoxicity (i.e., % killed cells). Untreated and untransduced cells served as controls. Data represent mean values ± standard error of the mean (SEM) from nine wells in three independent experiments (*** = p<0.001 selected clone and treated cells vs. all controls).

To test whether the selected AAV capsid mutants allow for improved gene delivery to primary PymT breast cancer cells, we produced rAAV luciferase vectors displaying the selected peptides RGDLGLS, RGDMSRE, and DGLGRLV for further analysis. These vectors transduced primary PymT breast cancer cells up to 17.8-fold better than wild-type AAV-2 vectors, and up to 3.500-fold better than vectors displaying an unselected random peptide (VRRPRFW) (Figure 2A). In contrast, HeLa cervical cancer cells, 3T3 mouse fibroblasts and primary mouse hepatocytes were not permissive for transduction with the selected capsid variants while they could be efficiently transduced with wild-type AAV transduction (data not shown), suggesting target specificity of the selected clones and supporting their use for targeting breast cancer cells *in vivo*. These findings were further corroborated by experiments using modified vectors harboring SR39, a derivative of the HSV-tk suicide gene [45], [51], [52]. Primary PymT breast cancer cells transduced by vectors with the RGDLGLS capsid insert showed strong cytotoxic effects upon ganciclovir treatment, whereas cells transduced with control vectors were almost resistant to ganciclovir (Figure 2B). Taken together, these findings suggest RGDLGLS-AAV as an interesting candidate for targeting therapeutic genes to breast cancer cells and demonstrate that our novel Ad5-free, PCR-based biopanning protocol allows for selection of targeted AAV vectors from random AAV display peptide libraries.

We therefore investigated whether the capsid mutants selected *in vitro* can target PymT breast tumors *in vivo*. AAV luciferase vectors displaying the selected peptides RGDLGLS, RGDMSRE, DGLGRLV, an unselected control peptide, or no peptide (wild-type AAV), respectively, were injected intravenously into female PymT mice bearing breast cancers. Analyses of reporter gene expression in tumor tissue revealed that none of the vectors mediated gene transduction in the tumor tissue (data not shown).

### Kinetics of circulating AAV peptide library particles and wild-type AAV are similar

**Figure 3:**
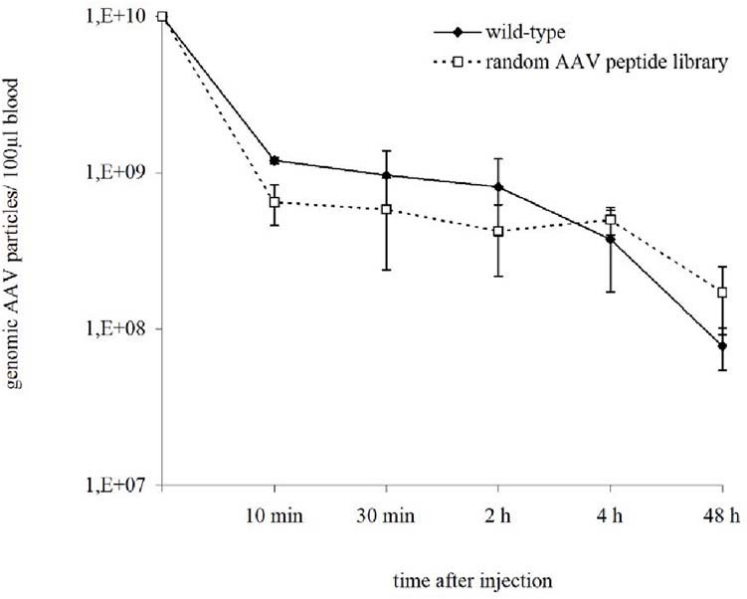
Kinetics of circulating AAV peptide library particles is similar to wild-type AAV. A random X_7_ peptide library or wild-type AAV-2 viruses were injected intravenously at 1×10^10^ vg per mouse. Blood samples were collected after indicated time points and the amount of circulating viral particles in the serum was determined by real-time PCR. Data represent mean values from n = 3 mice per group, analyzed in triplicates ± SD.

Based on the negative finding above, we hypothesized that selection under *in vivo* conditions is needed to enrich library clones that are able to bind cellular receptors in tumors, penetrate the tumor tissue and are internalized into tumor cells under physiological circulation conditions after intravenous administration. But we suspected that our novel PCR-based selection of AAV libraries may not be able to distinguish between library particles successfully internalized into target cells, and non-homing particles present in the circulation if the tissue is harvested too early after injection. To minimize the amount of circulating AAV library particles in our tissue samples at the time point of harvest, we analyzed the kinetics of circulating AAV library particles. Therefore, AAV were injected intravenously at 1×10^10^ vg per mouse, blood samples were collected at various time points, and the amount of circulating particles in the serum was quantified by real-time PCR. Clearance rates were comparable in AAV library particles and wild-type viruses (Figure 3). The amount of circulating genomes decreased in a straight proportional manner. We therefore decided to harvest tissues in AAV library selections 48 hours after virus administration.

### In vivo selection of AAV display peptide libraries in tumor tissue results in enrichment of distinct peptide inserts

Two technical approaches were chosen for AAV library selections *in vivo* (Figure 1, pathways B and C). Secondary libraries were produced and analyzed as for *in vitro* selections. Genomic titers of selected libraries allowed for injection of 2×10^8^ vg per mouse in selection rounds 2–4 (data not shown). After 4 rounds of selection, sequencing revealed the enrichment of serine and glycine-rich peptide motifs and repetition of several single clones. In particular, the motifs GGLSGXS and ESGXXXX, and the single clones EYRDSSG, QMSGGVA, EEPALRA, as well as APTLGLS were enriched during *“in vivo/ex vivo”* selections (Table 2). In a separate approach, we performed 2 further rounds of *ex vivo* selection with libraries pre-selected for 2 rounds on PymT cells *in vitro* (like in Figure 1, pathway A). Here, the only remaining clone following the *in vivo* part of this selection displayed the peptide DLGSARA (Table 2). During *in vivo* selections (Figure 1, pathway C), the peptide motifs enriched during four rounds of selection were XXSGVGS, GEARXXA, and SGNSGAA, as well as SSGSGGA and ESGIWVA (Table 2). The clones SGNSGAA and SSGSGGA shared the similar sequence pattern SSG or SGG, respectively, which also occurred in the EYRDSSG and QMSGGVA clones enriched during *ex vivo* selection. The motif ESGXXXX was highly enriched in both *in vivo/ex vivo* and pure *in vivo* selections. These data suggest that AAV library selection under circulation conditions is feasible and causes enrichment of a distinct pattern of displayed peptides after multiple rounds of biopanning. Therefore, we decided to evaluate *in vivo* gene transduction for all enriched clones.

**Table 2:** Peptides enriched in tumor tissue after selection for tumor-homing AAV

Selection pathway	Peptide a	Charge pattern b	Frequency in selection round^ c^			
			round 1	round 2	round 3	round 4
**Pathway B**	**GGLSGVS**	yyxxyyx	-/6	-/7	1/22	7/41
	**GGLSGDS**	yyxxy-x	-/6	-/7	-/22	1/41
	**GSVSGSA**	yxyxyxy	-/6	-/7	-/22	1/41
	**EYRDSSG**	-y+-xxy	-/6	-/7	-/22	7/41
	**QMSGGVA**	xyxyyyy	-/6	-/7	-/22	1/41
	**ESGLSQS**	-xyyxxx	-/6	1/7	1/22	2/41
	**ESGIWVA**	-xyyyyy	-/6	-/7	1/22	2/41
	**EEPALRA**	--yyy+y	-/6	-/7	-/22	4/41
	**APTLGSP**	yyxyyxy	-/6	1/7	-/22	13/41
**Pathway B** d	**RGDLGLS**	+y-yyyx			5/16	-/10
	**DLGSARA**	-yyxy+y			2/16	10/10
	**DGLGRLV**	-yyy+yy			6/16	-/10
	**DLRGLAS**	-y+yyyx			1/16	-/10
	**DRSPLSL**	-+xyyxy			1/16	-/10
**Pathway C**	**AISGVGS**	yyxyyyx	-/6	1/15	2/24	2/32
	**DRSGVGS**	-+xyyyx	-/6	1/15	4/24	2/32
	**SISGVGS**	xyxyyyx	-/6	-/15	-/24	1/32
	**SEGRSGV**	x-y+xyy	-/6	-/15	-/24	1/32
	**GEARSRA**	y-y+x+y	-/6	-/15	-/24	1/32
	**GEARISA**	y-y+yxy	-/6	-/15	2/24	7/32
	**SGNSGAA**	xyxxyyy	-/6	1/15	4/24	8/32
	**SSGSGGA**	xxyxyyy	-/6	-/15	2/24	2/32
	**ESGIWVA**	-xyyyyy	-/6	-/15	-/24	2/32

a single letter code; only peptides occurring repetitively or sharing common sequence motifs are shown; shared amino acid patterns are highlighted in colored letters

b charge pattern of amino acid side chains: +, positively charged; − negatively charged; x, uncharged polar; y, nonpolar.

c observed frequency relative to overall number of sequenced clones

d pathway B subsequent to 2 rounds of *in vitro* selection as in pathway A

### Selected library-derived AAV transduce tumors in vivo

**Figure 4:**
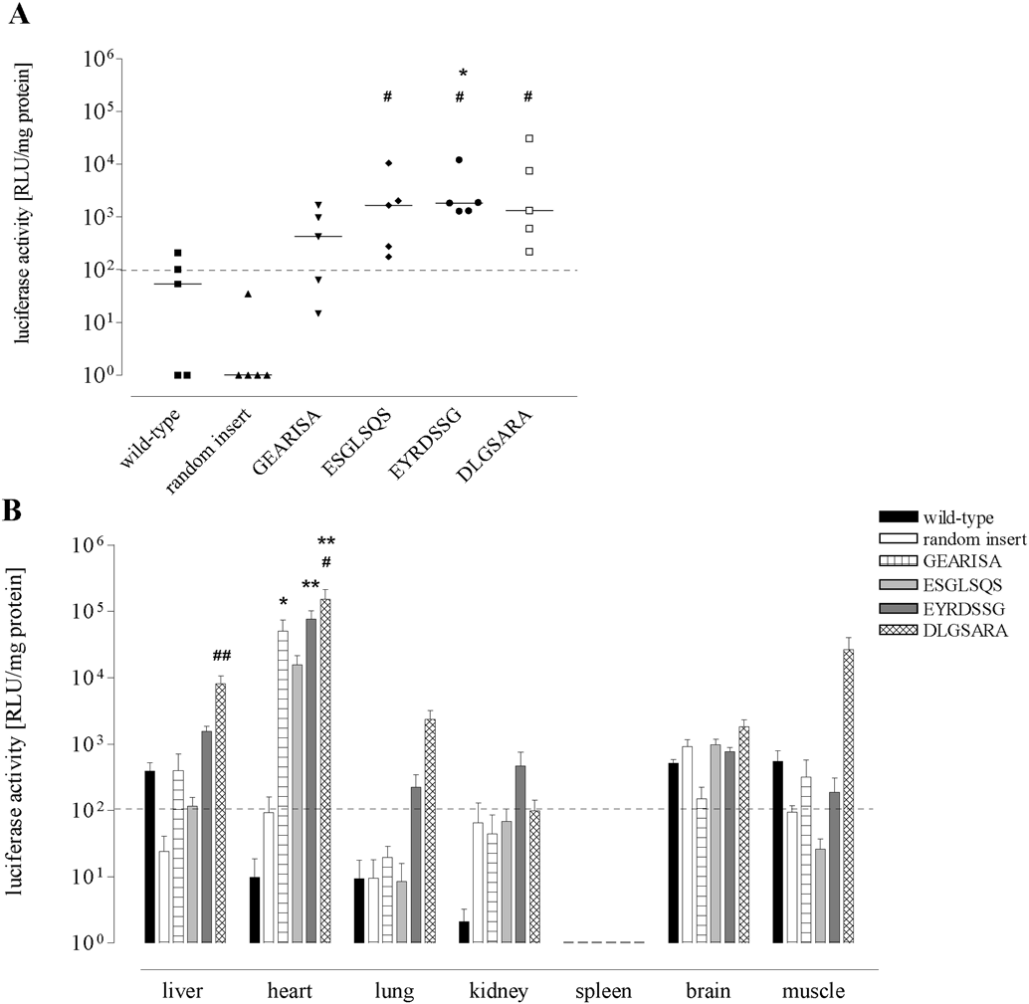
Gene delivery by AAV capsid mutants selected for breast cancer transduction *in vivo.* AAV luciferase vectors displaying selected peptides or controls (wild-type or VRRPRFW) were injected intravenously into female PymT tumor-bearing mice. After 8 d, representative tissues were harvested and luciferase activities were determined in individual tissues as relative light units (RLU) per mg protein. A: *In vivo* transduction of tumor tissue in PymT transgenic FVB mice by selected AAV mutants. Bars indicate the median, n = 5 mice per group. * p<0.05 targeted vectors vs. wild-type. # p<0.05 targeted vectors vs. random insert control. B: *In vivo* transduction of various non-cancerous tissues in PymT transgenic FVB mice by tumor-selected AAV mutants. The dotted line indicates the threshold beyond which luciferase expression data could be reliably delineated from background signal. Data represent mean values ± SEM, n = 5 mice per group. * p<0.05; ** p<0.01 targeted vectors vs. wild-type AAV-2. # p<0.05; ## p<0.01 targeted vectors vs. random insert control.

To assess whether the *in vivo*-selected AAV-2 vectors mediate gene expression in tumor tissue *in vivo*, we produced luciferase reporter vectors displaying the selected peptide sequences. All vectors titers ranged between 8×10^10^ to 7×10^11^ vg/ml (data not shown). Luciferase gene expression in the breast tumor tissue was evaluated 8 days after intravenous injection in tumor-bearing PymT mice in a screening experiment to assess which clones should be investigated in detail. Five of the clones (GEARISA, SGNSGAA, ESGLSQS, EYRDSSG, and DLGSARA) showed an increased transduction of the breast tumor tissue compared to wild-type AAV vectors, whereas unselected control vectors did not mediate any gene expression (data not shown). We chose the most promising vectors for further experiments in a larger group of animals (n = 5 mice per clone). Following intravenous injection, the selected clones transduced tumor tissue up to ∼275-fold more efficient compared to wild-type AAV vectors (Figure 4A). To further investigate the specificity of selected AAV capsid mutants, luciferase expression in several control organs was evaluated (Figure 4B). Moderate de-targeting from the liver by clone ESGLSQS and the unselected control was observed, whereas clones GEARISA and EYRDSSG transduced the liver in a manner comparable to wild-type AAV. DLGSARA gene transduction in liver tissue was significantly increased compared to the unselected control vector. Further, we found a strongly enhanced cardiac luciferase expression for all clones, being statistically significant for GEARISA, EYRDSSG and DLGSARA, and a weakly enhanced cardiac transduction of the unselected control vector compared to wild-type AAV. In regard to tissue specificity, the ESGLSQS clone had the most favorable profile as it transduced tumor tissue but not the liver. However, cardiac gene transduction was seen for this as for almost all the other clones as well. Reproduction of *in vivo* gene transduction with independent vector preparations for DLGSARA and ESGLSQS precisely confirmed our results (data not shown).

### In vivo selection of AAV capsids targeting lung tissue

To address the question whether the organ transduction pattern of clones obtained by *in vivo* AAV library screenings depends on the target tissue the library was selected for, we also selected AAV libraries for preferential homing into lung tissue. The screening was done along the lines of the tumor targeting approach (Figure 1, pathway C). We varied the time of library circulation before tissue harvest in the first round (5 minutes and 2 days, respectively) in two independent approaches. For both selections, circulation time was increased to 6 days in selection rounds 2–4. After 4 rounds of *in vivo* selection for both approaches, sequencing of the peptide insert of the AAV clones recovered from the lung revealed a striking consensus sequence motif, PRSAD(D/L)(A/S) , which was enriched independently in both selection procedures (Table 3). These data show that *in vivo* selection of AAV libraries *in vivo* in distinct tissues yields distinct peptide inserts, suggesting tissue specificity of the selection process.

**Table 3:** Peptides enriched in lung tissue during *in vivo* selection for lung-homing AAV after four rounds of selection

Selection pathway	Peptide a	Charge pattern b
**5 minutes circulation**	**PRSADLA**	y+xy-yy
	**PRSADLA**	y+xy-yy
	**VRSAADI**	y+yyy-y
	**PRSTSDP**	y+xxx-y
	**PRSTSDP**	y+xxx-y
	**PRSVDLS**	y+xy-yx
	**RGDLGLS**	+y-yyyx
**2 days circulation**	**PRSADLA**	y+xy-yy
	**PRSADLA**	y+xy-yy
	**PRSADLA**	y+xy-yy
	**VRSAADI**	y+yyy-y
	**PRSTSDP**	y+xxx-y
	**PRSVDLS**	y+xy-yx
	**PRSVDLS**	y+xy-yx
	**PASADLA**	yyxy-yy
**Consensus motif**	**P R S A D (^D^/_L_) (^A^/_S_)**	

a single letter code; shared amino acid patterns are highlighted in red letters

b charge pattern of amino acid side chains: +, positively charged; − negatively charged; x, uncharged polar; y, nonpolar.

### AAV clones displaying the PRSAD(D/L)(A/S) motif transduce lung tissue in vivo after systemic administration

**Figure 5:**
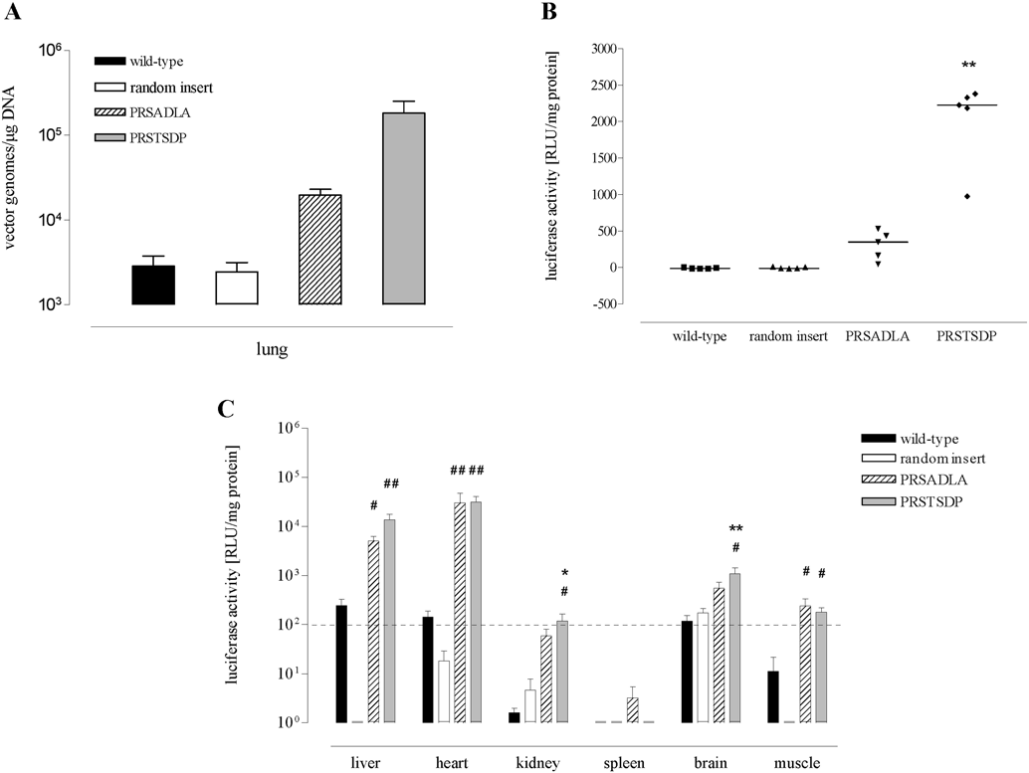
Targeting of AAV capsid mutants selected on murine lung tissue *in vivo.* AAV luciferase vectors displaying selected or control capsids (wild-type or random insert VRRPRFW) were injected intravenously into female FVB mice. Tissue was harvested after 8 or 28 d, respectively, and processed as indicated. A: Evaluation of lung homing. Lung tissue was harvested 8 days after vector injection and the amount of AAV genomes was determined by quantitative PCR. Data represent mean values from n = 3 mice per group, analyzed in triplicates ± SD. B: *In vivo* lung gene transfer by selected AAV after intravenous injection. Lung tissue was harvested 28 days after vector injection, and luciferase activity was determined as relative light units (RLU) per mg protein. Bars indicate the median value, n = 5 mice per group (** = p<0.001 targeted vectors vs. wild-type and random insert control). C: *In vivo* transduction of various tissues in mice by AAV library mutants selected for lung transduction. Tissues were harvested and luciferase activity was determined as in 5B. The dotted line indicates the threshold beyond which luciferase expression data could be reliably delineated from background signal. Data represent mean values ± SEM, n = 5 mice per group. * p<0.05; ** p<0.01 targeted vectors vs. wild-type AAV-2. # p<0.05; ## p<0.01 targeted vectors vs. random insert control.

Reporter gene vectors were made carrying the PRSTSDP and PRSADLA peptides or controls and gene transduction *in vivo* was evaluated. In a first step, we investigated whether the selected AAV capsid variants home to lung tissue more efficiently than AAV control vectors (wild-type or random insert capsids). Vectors were administered intravenously, and DNA was recovered from lung tissue after 8 days. Quantitative PCR of the CMV promoter region of the vectors revealed an up to 63-fold higher yield for the selected capsid variants compared to AAV-2 wild-type vectors and up to 74-fold higher yield compared to random control insert vectors (Figure 5A). Evaluation of luciferase expression in the lung 28 days after intravenous administration revealed a 35-fold and 233-fold increased transduction efficiency of PRSADLA and PRSTSDP, respectively, compared to wild-type AAV (Figure 5B). To determine the specificity of lung-targeted capsids, luciferase expression in several control organs was evaluated. Both selected clones showed higher gene transduction in liver, heart, kidney, brain, and muscle, compared to unselected controls (Figure 5C), suggesting that the cellular target bound by the selected vectors *in vivo* is ubiquitously rather than lung-specifically expressed.

## Discussion

Vector targeting *in vivo* is of paramount importance in gene therapy. For adeno-associated virus (AAV), this issue has been addressed by the insertion of peptide ligands into the vector capsid [6], [7], [8], [9], [10], [11], [12] or by exploiting the diversity of the various capsid serotypes [4], [5]. Despite considerable progress in this field, the availability of tissue-directed vectors for *in vivo* use is very limited, especially if delivered intravenously.

The screening of random AAV display peptide libraries is an innovative tool to select for vectors efficiently transducing any cell type of interest and has been described and validated for various cell types *in vitro* [14], [33], [34], [35], [36]. Most of the vectors described in these studies, however, have not been validated for *in vivo* use.

In our studies, library-derived vector capsids (displaying RGDLGLS, DGLGRLV) selected on primary murine breast cancer cells *in vitro* transduce the cell type they were selected on with high efficiency but fail to mediate breast cancer tissue directed gene transduction after systemic administration. As a consequence, we performed screenings of AAV libraries *in vivo* over multiple selection rounds after tail vein injection via the blood stream, using tumor and lung as target tissues. Several peptide clones were enriched in tumors and a clear-cut peptide sequence motif was recovered from the lung. While we achieved transduction of the target tissue by the selected vectors, we failed to achieve truly tissue-specific transgene expression. Therefore, except for liver transduction, most of our selected vectors have a tropism that is expanded to rather than specific for the tissue of interest compared to wild-type AAV-2. This may be due to several reasons: 1) the lack of tissue-specific receptors; 2) the expression of receptors conferring optimum transduction in several tissues, so capsids targeting receptors that are tissue-specific, but less efficient for transduction are not enriched; 3) superordinate (not receptor-dependent) factors influencing the selection process such as endothelial barriers, blood-derived factors, or extracellular matrix interactions. The first reason can be virtually excluded based on the overwhelming success rate of *in vivo* tissue targeting using phage display libraries [16], [17], [18], [19], [20], [21], [22], [23], [24], [25], [26], [27], [28], [29], [30], [31], [32]. Regarding the expression of non-tissue-specific receptors that are compatible with optimized AAV transduction, we think that two factors may play a role. Some of the selected peptides mediated transduction of several tissues with a clone-dependent transduction pattern, suggesting that the tropism is mediated by the targeting peptide. Especially for the lung-transducing vectors, the broad-spectrum tropism may also be due to the mechanism of library selection. Upon intravenous injection, virus capsids with optimized *in vivo* binding behavior may have been enriched in the lung irrespective of tissue specificity due to the first-pass effect after intravenous injection. These vectors may well be cell type-specific but not tissue-specific. They may be directed to endothelia in general, which is underlined by the fact that a similar capsid mutant (PRSVTVP) has been previously selected on primary endothelial cells *in vitro* [14]. This emphasizes the importance of the simultaneous negative selection in library screenings *in vivo* that can be achieved in tissues other than the lung. If the lung is the target of a screening, additional negative selection steps on endothelial cells prior to *in vivo* selection might alleviate some of the specificity challenges encountered in our targeting experiments. The second factor influencing the extended but unspecific tropism relates to the remarkable observation that as long as our selected vectors conferred any transgene expression *in vivo*, it invariably also occurred in the heart in addition to the target tissue. Heart expression of these vectors was even stronger than in wild-type AAV vectors. This is congruent with previous studies describing increased heart transduction upon modification of the VP3 region R484E/R588E [13] and peptide insertions at position R588 [14]. Our results in conjunction with the previously published data suggest that cardiac gene transduction may be mediated by a redistribution effect resulting from ablation of the endogenous tropism of the vector [13], [14], but clearly is also mediated by the design of the peptide insert as it varied from clone to clone. This might indicate that a capsid region close to the library insert at position R588 contributes to this tropism and that it may therefore be independent of the selected peptide sequence as such. Yet, biodistribution studies have not revealed increased heart homing by peptide insertion in this region [10], [11], which might in part be attributable to, or at least influenced by, a slightly differing insertion site (position N587 instead of R588). These findings re-emphasize that the gene expression profile mediated by a gene vector is not solely reflected by its biodistribution profile but also depends on factors like intracellular processing, promoter activity and vector clearance mechanisms.

Perabo *et al.* recently described that peptides containing a net negative charge and inserted at the AAV capsid position 587 are prone to confer a heparin sulfate proteoglycan non-binding phenotype which correlates with liver and spleen de-targeting in mice [15]. However, in our screenings we also isolated negatively charged peptides such as EYRDSSG which retained strong liver transduction. This may suggest that such selected capsid variants in our study indeed do not bind to heparin sulfate but rather to a ubiquitously expressed alternative receptor. In turn, we also isolated capsid mutants such as ESGLSQS which de-target from the liver compared to wild-type AAV, contradicting the assumption that additional mutations outside of the peptide insertion site at position 588 may be required to achieve this de-targeting effect.

Despite its obvious limitations, *in vivo* screening of AAV-2 libraries allows for selection of vectors with an extended tropism towards the tissue of interest. Vectors targeting the endothelial cell layer *in vivo* might be used to deliver anti-angiogenic genes such as endostatin in order to block neovascularization and tumor growth [53]. Furthermore, vectors transducing various organs might be useful when expression in tissues other than the primary target is desirable or uncritical as it has been performed by expression of the SOD gene delivered by adenovirus to protect lung tissue against radiation-induced fibrosis [54]. Compared to AAV vectors like AAV6 transducing lung tissue after nasal aspiration [55], vectors like the ones presented here which transduce lung tissue after intravenous injection, may also target other cell types such as lung endothelia and may therefore be considered as a valuable addition to the arsenal of lung-directed gene vectors. Finally, the tumor-directed vectors displaying the ESGLSQS peptide that mediates AAV transduction of breast cancer tissue *in vivo* and AAV de-targeting from the liver may further be optimized by using tumor specific expression systems such as the hTERT promoter [56], [57].

In previous work on AAV libraries, internalized virus particles were amplified by adenoviral delivery of helper proteins [14], [33], [34], [35]. However, the pathogenicity of adenovirus impedes the use of this strategy for *in vivo* selections, especially if it has to be administered systemically. Furthermore, the helper-dependent selection requires a near to complete adenoviral infection of the target tissue in order to maximize the amount of clones that can be amplified. Using currently available helper viruses, this can not be achieved in all organs and tissues *in vivo*, particularly in light of the fact that many target tissues or cell types are not susceptible to adenoviral infection after systemic administration. In addition, it is not clear (and at least in the tumor tissue used in our study very questionable) if AAV can replicate in mouse tissue efficiently. Our novel selection method addresses these points. Relevant parts of the genomes of tissue-targeted library viruses are amplified via nested PCR. We distinguished between three alternative selection pathways, all of which are based on the amplification and enrichment of tissue-homed AAV library particles by PCR during the selection process. Pathway A is a cell-based *in vitro* selection approach in which genomes of internalized library viruses are amplified while non-internalized viral particles are eliminated. Although our PCR selection protocol does not exclusively force the selection for AAV capsids that mediate gene expression, we demonstrate the functionality of this technical approach in that clones sharing a common peptide motif (RGDXXXX) were recovered by screening on primary murine breast cancer cells and conferred efficient transduction of these cells, even in a cytotoxic suicide gene transfer approach. Similar peptide motifs have been selected on PC3 prostate carcinoma cells [34] and M07e human leukemic megakaryocytic cells [33] by adenovirus-based selection. Incorporation of the RGD sequence into the viral capsid can target the vector to integrins, which are widely expressed on several cell types [6], [8], [58]. This suggests that AAV clones with an RGD-containing peptide insert might target via the integrin class of receptors [59]. The RGD integrin recognition sequence is also present in the so-called RGD4C-peptide which binds αvß3 or αvß5 integrins. This peptide homes to tumors *in vivo* after systemic administration and has therefore been widely used to target cellular integrins expressed in the tumor tissue of xenograft mouse models [17], [60], [45], [61]. It may therefore be tempting to speculate that the RGD-displaying AAV clones presented here might, like the RGD4C-peptide, also target tumor cells via the α_V_ class of integrins. However, we consider this unlikely because the clones did not show preferential homing to tumor tissue *in vivo* and RGD as a tripeptide sequence by no means specifically binds to this class but also to other classes of integrins. In fact, 12 of the 20 currently known integrins recognize a certain RGD-containing sequence as their ligand [59].

Pathways B and C aimed at selection of virus capsid variants after systemic administration of AAV libraries *in vivo*. These pathways take into account several hurdles that limit viral receptor targeting under *in vivo* conditions. Thus, tissue homing particles with weak or unspecific binding capacities toward their targets are eliminated by host clearance mechanisms or by homing to other tissues, although the screening process does not guarantee that the selected clones confer gene expression in addition of homing to the target tissue. Upon using selection pathways A, B, or C for tumor targeting, the enriched peptide sequences varied depending on the respective selection pathway, indicating that the most suitable screening conditions may have to be evaluated for each individual target tissue.

Compared to the previous work in which AAV libraries were selected *in vitro* on various target cells, these results are a significant step forward and profoundly expand our knowledge on the mechanisms involved in the *in vivo* gene transduction of vectors derived from biopanning of AAV peptide libraries. In addition, they overcome some of the limitations observed in a recent report by Grimm *et al.* [37]. In this pivotal work, AAV libraries were selected *in vivo* based on topical application to the airways (as opposed to systemic administration like in our study). Consequently, while two selected capsid mutants (NSSRDLG, MVNNFEW) in this study mediated gene transfer to certain cell types in the lung tissue after administration by inhalation, they failed to mediate vector retargeting after systemic administration. Interestingly, the NSSRDLG peptide insert described in this study was also described in previous studies on other cell types [14], [37] but also overlaps with the DLGSARA insert isolated from breast cancer tissue in the work presented here. Yet, both peptides probably bind to different receptors as the *in vivo* tropism seems to be very different which indicates that, at least for AAV, the whole peptide sequence rather than parts of a sequence motif has to be considered to evaluate its tropism.

In addition, the two selections (in Grimm *et al.* [37] and in our study) were done in a different AAV backbone context. Of note, in the work by Grimm *et al.* [37], the diversity of recovered AAV after two rounds of selection was restricted to one clone, presumably due to inefficient amplification and rescue of clones using adenoviral helper functions. Such outcome might change upon applying our novel PCR amplification protocol. Furthermore, novel AAV library principles like sequence evolution by error-prone PCR [62] and DNA shuffling [63] might enhance specificity and efficiency if used for *in vivo* selection.

This is the first report of *in vivo* biopanning with a systemically administered random AAV peptide library over multiple selection rounds. We show that vectors displaying *in vivo*-selected peptides have a significantly improved transduction profile in breast cancer or lung tissues after systemic administration. These findings demonstrate the superiority of AAV clones selected *in vivo* over clones selected *in vitro,* as long as *in vivo* transduction is required. Unintended cardiac transduction by selected clones remains the major limitation to be addressed in subsequent studies, *e.g.*, by further functional mapping of the capsid. Our findings broaden the understanding of AAV transduction profiles *in vivo*, the functionality of random AAV display peptide libraries and, even beyond the specific targets tumor and lung, are an important step in the development of targeted AAV gene vectors.
